# Long-Lasting Insecticidal Nets Incorporating Piperonyl Butoxide Reduce the Risk of Malaria in Children in Western Kenya: A Cluster Randomized Controlled Trial

**DOI:** 10.4269/ajtmh.20-1069

**Published:** 2021-06-14

**Authors:** Noboru Minakawa, James O. Kongere, George O. Sonye, Peter A. Lutiali, Beatrice Awuor, Hitoshi Kawada, Rie Isozumi, Kyoko Futami

**Affiliations:** 1 Institute of Tropical Medicine (NEKKEN), Nagasaki University, Nagasaki, Japan;; 2 Kenya Medical Research Institute, Nairobi, Kenya;; 3 Center for Research in Tropical Medicine and Community Development (CRTMCD), Nairobi, Kenya;; 4 Ability to Solve by Knowledge Project, Mbita, Kenya

## Abstract

Malaria vectors have acquired an enzyme that metabolizes pyrethroids. To tackle this problem, we evaluated long-lasting insecticidal nets incorporating piperonyl butoxide (PBO-LLINs) with a community-based cluster randomized control trial in western Kenya. The primary endpoints were anopheline density and *Plasmodium falciparum* polymerase chain reaction (PCR)-positive prevalence (PCRpfPR) of children aged 7 months to 10 years. Four clusters were randomly selected for each of the treatment and control arms (eight clusters in total) from 12 clusters, and PBO-LLINs and standard LLINs were distributed in February 2011 to 982 and 1,028 houses for treatment and control arms, respectively. Entomological surveys targeted 20 houses in each cluster, and epidemiological surveys targeted 150 children. Cluster-level permutation tests evaluated the effectiveness using the fitted values from individual level regression models adjusted for baseline. Bootstrapping estimated 95% confidence intervals (CIs). The medians of anophelines per house were 1.4 (interquartile range [IQR]: 2.3) and 3.4 (IQR: 3.7) in the intervention and control arms after 3 months, and 0.4 (IQR: 0.2) and 1.6 (IQR: 0.5) after 10 months, respectively. The differences were –2.5 (95% CI: –6.4 to –0.6) and –1.3 (95% CI: –2.0 to –0.7), respectively. The datasets of 861 and 775 children were analyzed in two epidemiological surveys. The median PCRpfPRs were 25% (IQR: 11%) in the intervention arm and 52% (IQR: 11%) in the control arm after 5 months and 33% (IQR: 11%) and 45% (IQR: 5%) after 12 months. The PCRpfPR ratios were 0.67 (95% CI: 0.38, 0.91) and 0.74 (95% CI: 0.53, 0.90), respectively. We confirmed the superiority of PBO-LLINs.

## INTRODUCTION

Because an effective vaccine is not available for malaria, targeting vectors is an efficient way to reduce parasite infection. Among a variety of vector control tools, insecticide-treated nets have been widely used since the early 2000s.^1–3^ As a result, the infection prevalence in endemic Africa halved between 2000 and 2015.^4,5^ However, the pace of reduction has stalled in recent years,^6,7^ and the current situation is far from realizing elimination. A significant change in the current control strategy is needed to push forward the efforts for malaria elimination.

The rapid expansion of vectors resistant to pyrethroid insecticides partially explains the slowing pace of reduction. Modeling based on the results of meta-analyses indicates that even low levels of resistance are able to increase the incidence of malaria.^8^ Malaria vectors have developed two main resistance mechanisms, target site resistance and metabolic resistance.^9^ The former resistance is related to the knockdown resistance (*kdr*) within the voltage-gated sodium channel gene; specifically, a point mutation at 1014L (L1014F or L1014S) causes insensitivity to pyrethroid insecticides.^10^ Metabolic resistance is mediated by the enhanced activity of one or more enzymes (cytochrome P450s) that metabolize pyrethroid insecticides.^11,12^

To inhibit the enzymatic activity related to metabolic resistance, long-lasting insecticidal nets (LLINs) incorporating piperonyl butoxide (PBO) have been developed.^13^ Several studies evaluated the effects of PBO on vectors under semifield conditions using experimental huts.^14–18^ A systematic review revealed that PBO-LLINs increase mosquito mortality by 84% compared with standard LLINs in highly pyrethroid-resistant areas.^19^

Two epidemiological studies have reported the effectiveness of PBO-LLINs on reducing infection risk. A cluster randomized controlled trial (cRCT) in Tanzania showed that after 9 months the PBO-LLINs reduce *Plasmodium falciparum*–positive prevalence in children versus standard LLINs based on a rapid diagnostic test (RDT).^20^ In Uganda, a cRCT based on microscopy also reported that parasite prevalence was lower in areas covered with PBO-LLINs.^21^ *Anopheles gambiae* s.s. with a high level of *kdr* resistance was predominant in the Tanzania study site, and there was evidence of the existence of a metabolic-resistant population.^22,23^ *Anopheles gambiae* s.s. with high *kdr* resistance was also predominant in Uganda, and the metabolic resistance was moderate among the vector populations.^21,24^ Because PBO-LLINs were developed to control vectors with metabolic resistance, it is important to determine the effectiveness of PBO-LLINs in an area where a metabolic-resistant vector population is predominant.

We conducted a community-based cRCT to evaluate PBO-LLINs in an area where *An. arabiensis* and *An. funestus* s.s. with metabolic resistance likely predominate.^25–30^ The primary endpoints were the density of vectors and a polymerase chain reaction (PCR)-based assay of *P. falciparum* positive prevalence (PCRpfPR) of children aged from 7 months to 10 years old. Instead of RDT and microscopy used in the previous epidemiological studies, we used PCR for the primary endpoint because it has higher sensitivity and specificity. Although the qualities of RDTs have improved, including the RDT (CareStart Malaria HrP2/pLDH (pf/pan) Combo, DiaSys, Wokingham, Berkshire, United Kingdom) used in the Tanzania study, the detectability of parasites decreases with a reduction in parasite density.^31,32^ Because field evaluation studies of LLINs target mainly asymptomatic individuals in natural populations, PCR becomes more reliable in the field than RDTs and microscopy.^33–35^ The secondary endpoints were RDT *P. falciparum*–positive prevalence (RDTpfPR) and hemoglobin (Hb) concentration.

## MATERIALS AND METHODS

### Study area.

**Figure 1.**
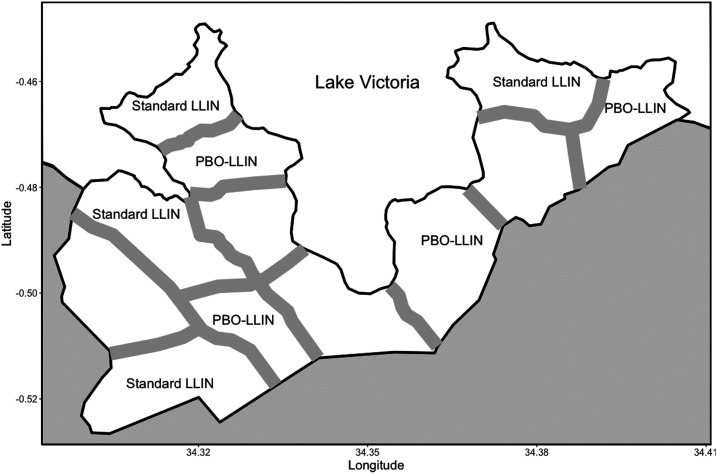
A map showing the boundaries and buffer zones between the intervention and control clusters in the study area.

The study area was Gembe East of Homa Bay County in western Kenya (Figure 1). The total land area was approximately 46 km^2^, and the coordinates of the geographic midpoint were 0°30′24″ S and 34°20′48″ E. The area was divided into 12 clusters based on 14 villages or communities. The mean area of the clusters was 3.8 km^2^ (SD = 0.86). We selected four clusters for PBO-LLIN intervention and four clusters for control, allocating computer-generated random numbers to the clusters. The remaining four clusters were used for a separate study of mosquito nets covering house ceilings,^28^ and a total of eight clusters were used for this study. We modified the historical community boundaries to create a “fried-egg” design based on the distribution of children found in the preliminary study.^28^ Although houses were scarce around the boundaries, buffer zones (300 m) were established to minimize a spillover effect between clusters based on the flight distance of vectors.^36,37^ Most houses are constructed with a stick framework plastered with a mixture of mud and cow dung and a corrugated iron roof. The majority of residents belong to the Luo ethnic group. Although Dholuo is the main language spoken, most residents speak English and Kiswahili. The main income sources are fishing, traditional small-scale farming, and cattle breeding.^28^

The species of malaria vectors recorded from this study area are *Anopheles arabiensis, An. gambiae* s.s., and *Anopheles funestus* s.s. When the former two species are grouped as *Anopheles gambiae* s.l., a study in the same area reported that nearly 90% of *An. gambiae* s.l. collected with the pyrethrum spray catch method (PSC) were *An. arabiensis*.^27^ The preliminary study found that 25% of anopheline mosquitoes collected with PSC were *An. funestus* s.l.,^28^ and a study in an adjacent area reported that nearly all *An. funestus* s.l. individuals collected indoors were *An. funestus* s.s.^29^ A small number of potential minor vector species (*Anopheles rivulorum*) belonging to *An. funestus* s.l. was also reported.^38^

A study on insecticide resistance found that more than 80% of field collected *An. gambiae* s.s. had homozygous L1014S mutations, whereas no single mutation at L1014S was found from *An. funestus* s.s. and *An. arabiensis* individuals collected in this study area.^25^ In an experiment of LD_50_ with mosquitoes collected from the study area, a topical application of PBO (1.25 µg) enhances the susceptibility of the latter two species to permethrin 22 to 69 times, whereas the enhancement for *An. gambiae* was 2 to 7 times.^26^ These results indicate that the metabolic-resistant vector population was predominant in the study area. Indoor residual spray was not implemented in the area before or during the present study. To protect mainly infants and pregnant women, LLINs had been distributed at health facilities.

### Preintervention survey (baseline survey).

For an entomological baseline we used the data from a sentinel surveillance between April 2009 and February 2011. Indoor resting mosquitoes were collected every 2 weeks from 10 sentinel houses within each cluster (80 in total) in the morning hours (6:30–10:00) using PSC. Details of the sentinel surveillance are described in published studies.^27,28^

**Figure 2.**
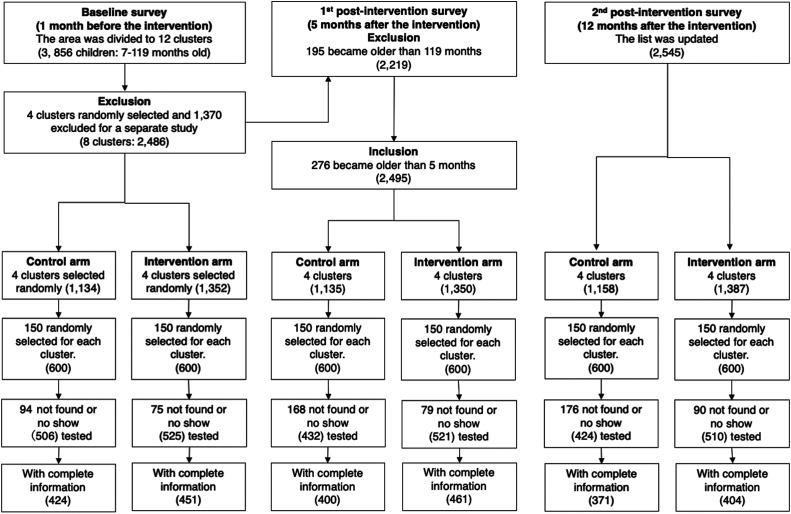
A flow chart and trial profile for the epidemiological surveys.

Before the epidemiological baseline survey, we held a series of meetings with the local chiefs, village elders, and district medical officers in early January 2011 and explained to them the goals of this study. Trained field assistants visited each house and recorded the number of residents, their ages and genders, the number of bed nets, and the geographic coordinates with a GPS (Garmin, Olathe, KS). From the baseline data, we listed children from 7 months to 10 years old. Then, we selected 150 children from the list for each cluster, allocating computer-generated random numbers to all eligible children (Figure 2). The preliminary study in 2010 estimated an intraclass correlation coefficient (ICC) of 0.053 based on RDTpfPR.^28^ We expected a 50% reduction of PCRpfPR in the treatment arm. As the RDTpfPR in the study area was 48% in the preliminary study,^28^ we expected PCRpfPR of 24% in the treatment arm, assuming the discrepancy between PCRpfPR and RDTpfPR was negligible for sample size calculation. With 80% power and an alpha of 0.05, the estimated cluster size was 116 children. We inflated the sample size to 150 because of anticipated dropouts.

Trained field assistants visited the households of the selected children, explained the study to their caretakers, and obtained informed written consent. The field assistants informed the caretakers of the primary school and community center testing locations and dates, and recorded information to assess socioeconomic status (SES). SES for each household was estimated using a composite household material wealth index based on the possession of various consumer goods, house construction, toilet and water access, and livestock.^39,40^ A numerical score was assigned to each household using multiple corresponding analysis. The continuous measures were then divided into tertiles to obtain a rough proxy of SES.

Within a few days after consent, we invited the selected children and their caretakers to a testing center established within each cluster. The axillary temperature of each child was measured, and a finger prick blood sample was taken to conduct RDT (Paracheck-Pf, Orchard Biomedical System, Goa, India) for detecting *P. falciparum* infection and to measure Hb concentration (g/dL) using a portable Hb photometer (Hemocue, Angelholm, Sweden). Two persons confirmed the results of the RDTs. Blood was also drawn into a 20-µL capillary tube (Thermo Fisher Scientific, Waltham, MA) to standardize the blood volume and was preserved on a filter paper (Whatman ET31 Chr, Whatman International, Maidstone, United Kingdom). Later, the sampled blood was examined to detect *P. falciparum* using PCR. Artemether-lumefantrine was given to each child who had a positive RDT and body temperature >37.5°C. Children with Hb concentration <11.0 g/dL were given iron supplementation. Some children whose symptoms did not follow these criteria were also given the treatment based on the WHO guideline and diagnosis by a clinician.^41^ While waiting for a RDT result, caretakers were interviewed on whether their children slept under an LLIN the previous night, a standard protocol to assess LLIN use.^42–48^ A study in an adjacent area found that the results from interviews for LLIN use were similar to those from direct observations in the early morning.^45^ They were also interviewed on whether children slept on a bed or nonbed sleeping location.^45,47^

### Intervention.

In February 2011, field assistants visited all listed houses and delivered enough PBO-LLINs (OlysetPlus, Sumitomo Chemical, Tokyo, Japan) in the intervention arm based on the WHO recommendation of at least one LLIN for every two persons.^48^ For houses with an odd number of persons, we provided extra LLINs to ensure that all persons had access to a net (e.g., two nets for three persons, and three nets for five persons). In the control arm, we provided enough standard LLINs (OlysetNet, Sumitomo Chemical) to all houses. The two types of LLINs had the same color and shape and similar texture. Residents and field assistants were not told which was the PBO-LLIN, but they were distinguishable by a unique code on the label. We removed old LLINs from the houses and hung new ones with consent.

### Postintervention survey.

To compare entomological data between both arms, we used the data from 80 sentinel houses (10 for each cluster) during the period between March 2011 and May 2012. Because the sentinel houses were not randomly selected,^28^ we also conducted a cross-sectional survey with 25 randomly selected houses in each cluster in May 2011 at the end of the long rainy season. The preliminary study estimated that the number of anophelines per house was 4.3, and the between cluster coefficient of variance was 0.192 for anopheline mosquitoes.^29^ In an area where *An. gambiae* with a high level of *kdr* was predominant, the mosquito mortality rate by PBO-LLINs was 78%, whereas it was 44% with standard LLINs.^14^ Therefore, we expected 50% reduction from the density of 4.3 per house in the treatment arm. With the sample size of 25 and an alpha of 0.05, the power was 94%. All selected houses were made of mud walls and consisted of one room. Indoor resting female anophelines were sampled using PSC. The cross-sectional survey was repeated with newly selected houses in December 2011, at the end of the short rainy season. Sampled anophelines were divided to *An. gambiae* s.l. and *An. funestus* s.l. under the microscope, and their numbers were recorded. The sampled houses were selected allocating computer-generated random numbers to all houses listed in each survey.

We conducted the first postintervention epidemiological survey in July 2011, after the long rainy season, following the same procedure as the baseline survey. Before the survey, the same number of children were randomly selected from the list created in January 2011 (Figure 2). We updated the list of children through another house survey in January 2012, after the short rainy season. The epidemiological survey was then repeated.

### Data management.

Three independent field teams collected entomological data, epidemiological data, and house-related data including LLINs. The data were recorded on paper forms. Two persons converted the data to a digitized form, and the data were independently verified. When discrepancies or missing data were found, staff were sent back to the field to confirm or re-collect data if possible. All houses, children, and LLINs were coded, and the finalized data were stored in a database in Nagasaki University for analyses and security.

### Statistical analysis.

The effectiveness of PBO-LLINs on the entomological endpoint was evaluated comparing the postintervention sentinel data between the two arms based on cluster-level summaries. We used a two-stage procedure that is able to increase statistical power adjusting the variability of baseline data among the clusters.^49,50^ This approach is particularly useful when the number of clusters is small. In the first stage, we used a regression model to obtain a residual of each cluster that was adjusted for the individual level preintervention baseline data. We first considered a Poisson regression model using R with the package lme4 because of count data.^51,52^ When data were overdispersed, a negative binomial model was applied. We also considered houses and sampling dates as potential random factors because the same houses were sampled every 2 weeks in the sentinel surveillance. Using the fitted model, a fitted value was summarized for each cluster. In the second stage the difference between the fitted value and the observed value was obtained for each cluster, and we applied a permutation test based on the ranks for evaluating the median difference between the two groups with the R package coin.^53^ To estimate a cluster level effect size and 95% confidence interval (CI), we used bootstrapping (the bias-corrected accelerated bootstrap percentile) with the R package boot.^54^ Bootstrapping is more suitable than permutation for estimating effect size and CI because these values do not assume that a null hypothesis is true.^55,56^ The two-stage procedure was also applied to the cross-sectional entomological data incorporating the preintervention sentinel data as a baseline. We analyzed data of each of the two taxonomic groups separately and combined data as anopheline.

Similarly, we applied the two-stage procedure for evaluating the effectiveness of PBO-LLINs on the primary epidemiological endpoint (PCRpfPR) and the secondary endpoints (RDTpfPR and Hb concentration). In the first stage, a logistic regression model was used for PCRpfPR and RDTpfPR. Although confounders were not available in the entomological analyses besides the baseline data, the epidemiological analyses included age, bed net use, sleeping location, SES, and the baseline prevalence data. Permutation tests were used to compare the prevalence ratio and absolute difference between the two groups. Bootstrapping was used to estimate the effect sizes and 95% CIs. A normal linear regression model was used for Hb concentration including the same covariates. We evaluated the absolute difference in Hb concentration between the two groups and estimated the effect size and 95% CIs.

### Ethics.

This trial was approved by the Ethics Committees of the Kenya Medical Research Institute (SSC No. 1310 and 2131) and Nagasaki University (No. 10121655-2). The study is registered with UMIN Clinical Trials Registry (UMIN000019971).

## RESULTS

### Baseline survey.

The house survey in early January 2011 recorded 3,352 houses and 12,098 residents in the study area. The median number of houses per cluster was 276 (interquartile range [IQR] = 75), and the mean number of residents per house was 3.6 (SD = 1.8). Ages were confirmed for 11,125 residents, and 3,900 (35%) of them were 7 months to 10 years old. As 44 children were later excluded because their houses were within the buffer zones, the target population became 3,856. After the random selection of four clusters for each of the intervention and control arms, the total number of houses became 2,176. The total number of eligible children was 2,486, and the median number of children per cluster was 300 (IQR = 73) in the baseline survey (Figure 2).

**Figure 3.**
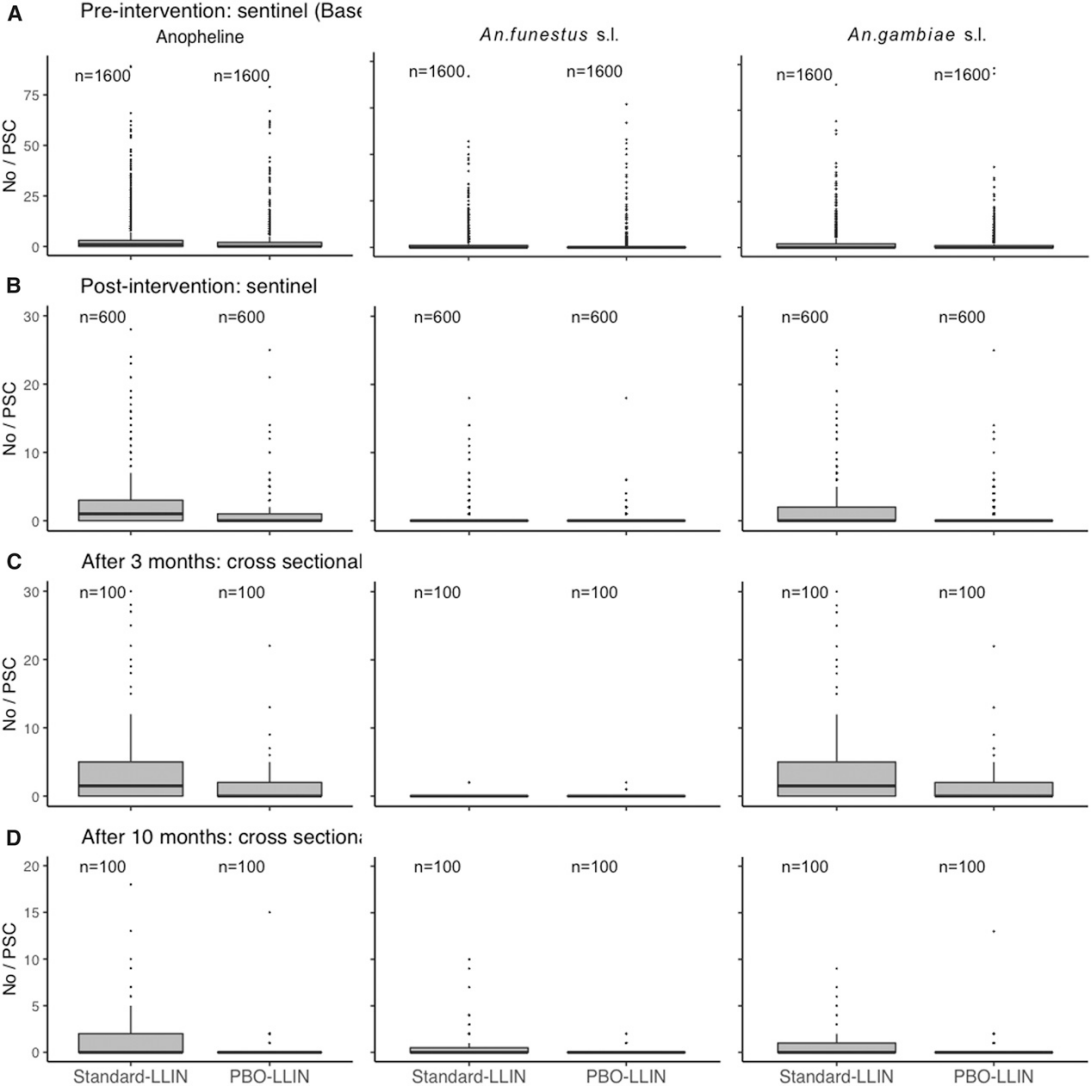
Vector densities from the sentinel house mosquito survey and two postintervention cross-sectional surveys.

The baseline entomological survey collected 10,671 anopheline mosquitoes from 3,200 PSCs at 80 sentinel houses in the randomly selected eight clusters during the period between April 2009 and February 2011. Of them, 4,466 (42%) were *An. funestus* s.l., and 6,205 (58%) were *Anopheles gambiae* s.l. The median number of anophelines per sample was 1 (IQR = 3), and those of *An. funestus* s.l. and *Anopheles gambiae* s.l. were 0 (IQR = 0) and 0 (IQR = 2), respectively (Figure 3).

In the epidemiological survey, 169 (14%) children did not show up at the testing centers, and we tested 1,031 (86%) of 1,200 randomly selected children for *P. falciparum* infection (Figure 2). We excluded incomplete data of 156 children and analyzed the remaining data from 875 (73%) children. The individual level PCRpfPR and RDTpfPR were 60% and 58%, respectively (Table 1). The individual level mean Hb concentration was 10.32 (SD = 1.90). The proportion of children who used bed nets the previous night was 55%.

**Table 1:** Individual-level summary statistics of the variables from the epidemiological baseline (preintervention) survey and two postintervention surveys

	Baseline		After 5 months		After 12 months	
Variable	Standard LLIN	PBO LLIN	Standard LLIN	PBO LLIN	Standard LLIN	PBO LLIN
Age (SD)	5.1 (3.2)	5.0 (3.0)	4.8 (2.5)	5.1 (2.9)	5.1 (2.8)	5.4 (2.9)
Gender						
Female	230 (54%)	237 (53%)	204 (51%)	234 (51%)	184 (50%)	209 (52%)
Male	194 (46%)	214 (47%)	196 (49%)	227 (49%)	187 (50%)	195 (48%)
Net use						
Used	230 (54%)	251 (56%)	360 (90%)	409 (89%)	340 (92%)	381 (94%)
Not used	194 (46%)	200 (44%)	40 (10%)	52 (11%)	31 (8%)	23 (6%)
SES						
Low	101 (24%)	116 (26%)	105 (26%)	141 (31%)	57 (15%)	46 (11%)
Middle	228 (54%)	205 (46%)	207 (52%)	212 (46%)	226 (61%)	231 (57%)
High	95 (22%)	130 (29%)	88 (22%)	108 (23%)	88 (24%)	127 (31%)
Sleeping location						
Bed	140 (33%)	124 (28%)	136 (34%)	142 (31%)	125 (34%)	115 (29%)
Nonbed	284 (67%)	327 (73%)	264 (66%)	319 (69%)	246 (66%)	289 (72%)
PCR						
Negative	174 (41%)	176 (39%)	221 (55%)	320 (69%)	198 (53%)	264 (65%)
Positive	250 (59%)	275 (61%)	179 (45%)	141 (31%)	173 (47%)	140 (35%)
RDT						
Negative	190 (45%)	175 (39%)	213 (53%)	325 (70%)	182 (49%)	255 (63%)
Positive	234 (55%)	276 (61%)	187 (47%)	136 (30%)	189 (51%)	149 (37%)
Hb g/dL (SD)	10.5 (1.8)	10.2 (2.0)	10.8 (1.7)	10.9 (1.7)	10.7 (1.6)	11.1 (1.7)
N	424	451	400	461	371	404

Hb = hemoglobin; LLIN = long-lasting insecticidal nets; PBO = piperonyl butoxide; PCR = polymerase chain reaction; RDT = rapid diagnostic test; SES = socioeconomic class.

### Intervention.

The number of target houses for LLIN distribution became 2,167 after excluding nine houses because the residents reportedly had migrated to other areas after the baseline survey. The residents of 92 houses were not available during the distribution in the intervention arm, and we could not provide LLINs to them. As a result, 1,959 PBO-LLINs were provided to 987 of 1,079 targeted houses (92%) in the intervention arm. The PBO-LLIN coverage was 2.0 nets per house and 1.9 persons per net when 92 houses without LLINs were excluded. Including the 92 houses, the figures became 1.8 nets per house and 2.1 persons per net. In the control arm, 2,112 standard LLINs were distributed to 1,028 of 1,088 target houses (95%). The standard LLIN coverage was 2.1 nets per house and 1.7 persons per net when the houses without new LLINs were excluded. When these houses were included, the coverage became 1.9 nets per house and 1.8 persons per net.

### Postintervention survey.

During the 15-month post-intervention period, 2,030 anopheline mosquitoes were collected from a total of 1,200 PSCs in 80 sentinel houses. Of them, 1,575 (78%) were *An. gambiae* s.l., and 455 (29%) were *An. funestus* s.l. (Figure 3). The adjusted differences in cluster level median density were –0.1, –0.5, and –1.4 for *An. funestus* s.l., *An. gambiae* s.l., and anopheline in the intervention arm, respectively (Table 2). The 95% CIs by bootstrapping indicated that the differences were significant for *An. gambiae* s.l. and anopheline; however, this was not the case for the permutation tests. The differences were not statistically significant for *An. funestus* s.l. with both permutation and bootstrapping.

**Table 2:** Effects of PBO-LLINs on vector densities. The effect sizes and 95% confidential intervals (95% CIs) were estimated with bootstrapping (the bias-corrected accelerated bootstrap percentile) based on cluster level median densities, and the differences between two arms were tested with permutational Wilcoxon rank sum tests.

	*An. funestus* s.l.			*An. gambiae* s.l.			*Anopheline* (Total)		
Variable	Standard LLIN	PBO LLIN	*P* value	Standard LLIN	PBO LLIN	*P* value	Standard LLIN	PBO-LLIN	*P* value
Preintervention sentinel survey: no/sample (IQR)	1.5 (2.9)	0.4 (0.1)		1.8 (1.7)	1.4 (1.2)		3.3 (4.6)	1.6 (2.2)	
Postintervention sentinel survey: no/sample (IQR)	0.4 (0.6)	0.1 (0.2)		1.8 (1.0)	0.5 (0.8)		2.5 (1.9)	0.6 (1.0)	
Unadj. difference	0 (ref)	–0.3 (–1.1 to 0.6)	0.149	0 (ref)	–1.3 (–4.1 to 0.6)	0.114	0 (ref)	–1.7 (–4.7 to 0.5)	0.114
Adj. difference	0 (ref)	–0.1 (–0.4 to 0.0)	0.149	0 (ref)	–0.5 (–0.7 to –0.1)	0.057	0 (ref)	–1.4 (–2.1 to –0.2)	0.057
Cross-sectional survey after 3 months: no/sample (IQR)	0.1 (0.1)	0.1 (0.1)		3.4 (3.7)	1.4 (2.3)		3.4 (3.7)	1.4 (2.3)	
Unadj. difference	0 (ref)	0.0 (–0.1 to 0.1)	0.91	0 (ref)	–2.0 (–12.7 to 3.1)	0.149	0 (ref)	–2.0 (–12.8 to 3.2)	0.149
Adj. difference	0 (ref)	0.0 (–0.1 to 0.1)	0.49	0 (ref)	–2.5 (–6.1 to –0.6)	0.029	0 (ref)	–2.5 (–6.4 to –0.6)	0.029
Cross-sectional survey after 10 months: no/sample (IQR)	0.8 (0.5)	0.1 (0.1)		0.5 (0.4)	0.2 (0.2)		1.6 (0.5)	0.4 (0.2)	
Unadj. difference	0 (ref)	–0.7 (–1.1 to –0.2)	0.029	0 (ref)	–0.3 (–1.6 to 0.0)	0.114	0 (ref)	–1.2 (–2.0 to –0.4)	0.029
Adj. difference	0 (ref)	–0.7 (–1.1 to –0.3)	0.029	0 (ref)	–0.4 (–1.4 to –0.2)	0.029	0 (ref)	–1.3 (–2.0 to –0.7)	0.029
*N*	4	4		4	4		4	4	

Adj = adjusted; CI = confidence level; IQR = interquartile range; LLIN = long-lasting insecticidal nets; PBO = piperonyl butoxide; Unadj = unadjusted.

In the cross-sectional entomological survey after 3 months of intervention, a total of 854 anophelines were collected from 200 PSCs. Of them, 846 (99%) were *An. gambiae* s.l., and 8 (1%) were *An. funestus* s.l. (Figure 3). The adjusted differences in density were 0, –2.5, and –2.5 for *An. funestus* s.l., *An. gambiae* s.l., and anopheline in the intervention arm, respectively (Table 2). Both permutation and bootstrapping indicated that the reductions were significant for *An. gambiae* s.l. and anopheline. The *An. funestus* s.l. result was likely due to the insufficient number.

After 10 months of intervention, the second entomological cross-sectional survey collected 200 anophelines including 109 (55%) *An. gambiae* s.l. and 91 (45%) *An. funestus* s.l. (Figure 3). The reductions in density were –0.7, –0.4, and –1.3 for *An. funestus* s.l.,* An. gambiae* s.l., and anopheline in the intervention arm (Table 2). Both permutation and bootstrapping confirmed that the reductions were statistically significant for the three cases.

In the first postintervention epidemiological survey, the number of eligible children was 2,495, and we analyzed the data from 861 (72%) of 1,200 randomly selected children after excluding children who did not show up or did not have complete data (Figure 2). The individual level PCRpfPR and RDTpfPR of the intervention arm were 31% and 30%, and those of the control arm were 45% and 47%, respectively (Table 1). Both cluster-level median PCRpfPR and RDTpfPR were 25% for the intervention arm, and those of the control arm were 52% (Table 3). When the analysis was adjusted for the baseline and the other covariates, in the intervention arm the PCRpfPR and RDTpfPR were reduced by 33% (95% CI: 9–62%) and 50% (95% CI: 24–62%), respectively. The adjusted absolute differences were –0.13% (95% CI: –32% to –5%) and –0.22% (95% CI: –32% to –5%) for PCRpfPR and RDTpfPR, respectively. The permutation tests also confirmed that the reductions were statistically significant for all adjusted PCRpfPR cases. Despite the significant effect sizes indicated by 95% CIs, the reductions in RDTpfPR were not significant with the permutation tests.

**Table 3:** Effects of PBO-LLINs on PCRpfPR, RDTpfPR, and Hb concentration (g/dL)

	After 5 months			After 12 months		
Variable	Standard LLIN	PBO LLIN	*P* value	Standard LLIN	PBO LLIN	*P* value
PCRpfPR: median (IQR)	0.52 (0.11)	0.25 (0.11)		0.45 (0.05)	0.33 (0.11)	
Unadjusted prevalence ratio	1 (ref)	0.50 (0.36 to 1.05)	0.149	1 (ref)	0.72 (0.53 to 1.08)	0.149
Adjusted prevalence ratio	1 (ref)	0.67 (0.38 to 0.91)	0.029	1 (ref)	0.74 (0.53 to 0.90)	0.029
Unadjusted prevalence difference	0 (ref)	−0.27 (–0.35 to 0.01)	0.114	0 (ref)	−0.12 (–0.26 to 0.03)	0.057
Adjusted prevalence difference	0 (ref)	−0.13 (–0.32 to −0.05)	0.029	0 (ref)	−0.11 (–0.21 to −0.03)	0.029
RDTpfPR: median (IQR)	0.52 (0.17)	0.25 (0.08)		0.50 (0.02)	0.37 (0.11)	
Unadjusted prevalence ratio	1 (ref)	0.57 (0.37 to 1.15)	0.114	1 (ref)	0.71 (0.44 to 1.02)	0.149
Adjusted prevalence ratio	1 (ref)	0.50 (0.38 to 0.76)	0.057	1 (ref)	0.71 (0.45 to 0.94)	0.029
Unadjusted prevalence difference	0 (ref)	−0.20 (–0.41 to −0.02)	0.114	0 (ref)	−0.14 (–0.28 to 0.01)	0.057
Adjusted prevalence difference	0 (ref)	−0.22 (–0.32 to −0.05)	0.057	0 (ref)	−0.15 (–0.27 to −0.03)	0.029
Hb concentration: median (IQR)	10.69 (0.16)	10.99 (0.45)		10.71 (0.13)	11.10 (0.10)	
Unadjusted difference	0 (ref)	0.20 (–0.23 to 0.60)	0.486	0 (ref)	0.39 (0.20 to 0.72)	0.029
Adjusted difference	0 (ref)	0.11 (–0.24 to 0.29)	0.343	0 (ref)	0.34 (0.15 to 0.48)	0.029
N	4	4		4	4	

CI = confidence level; Hb = hemoglobin; IQR = interquartile range; LLIN = long-lasting insecticidal nets; PBO = piperonyl butoxide; PCRpfPR = polymerase chain reaction* Plasmodium falciparum*–positive prevalence; RDT = rapid diagnostic test *Plasmodium falciparum*–positive prevalence. The effect sizes and 95% confidential intervals were estimated with bootstrapping (the bias-corrected accelerated bootstrap percentile) based on cluster level median ratios and differences. The cluster level differences between two arms were tested with permutational Wilcoxon rank-sum tests.

After 12 months of intervention, we analyzed the data of 775 (65%) of 1,200 randomly selected children (Figure 2). The individual-level PCRpfPR and RDTpfPR of the intervention arm were 35% and 37%, and those of the control arm were 47% and 51%, respectively (Table 1). In the cluster level, the PCRpfPR and RDTpfPR of the intervention arm were 33% and 37%, respectively, and those of the control arm were 45% and 50% (Table 3). The adjusted PCRpfPR and RDTpfPR were reduced by 26% (95% CI: 10–47%) and 29% (95% CI: 6–55%) in the intervention arm, respectively. The adjusted differences were –0.11% (95% CI: –21% to –3%) and –0.15% (95% CI: –27% to –3%) for PCRpfPR and RDTpfPR, respectively. The permutation tests confirmed that all reductions were statistically significant for both adjusted PCRpfPR and RDTpfPR cases.

Both individual-level and cluster-level Hb concentrations were higher in the intervention arm than the control arm after 5 and 12 months (Tables 1 and 3). Although the permutation test and CIs showed that the cluster level difference between the intervention and control arms was not statistically significant after 5 months of intervention, it became statistically significant with both permutation and bootstrapping after 12 months. The cluster-level median Hb concentration was greater in the intervention arm by 0.34 g/dL (95% CI: 0.15–0.48 g/dL) after 12 months (Table 3).

## DISCUSSION

The present study shows that PBO-LLINs are more effective than standard LLINs in reducing *P. falciparum* infection in children. The cluster-level adjusted PCRpfPR ratios were 0.67 and 0.74 at 5 and 12 months postintervention, respectively, when compared with standard LLINs. The RDT based ratios were 0.50 and 0.71 at 5- and 12-months postintervention. These values are almost the same as those from studies in Tanzania and Uganda where the ratios were 0.69 to 0.73 and 0.74, respectively.^20,21^ Including the present study, all three studies clearly show that the added effectiveness of PBO-LLINs was significant. As a result, the present study strengthens the previous findings.

Although it is not appropriate to compare the outcomes before and after the intervention because of temporal variations mainly caused by seasonal climate, the individual-level PCRpfPR was halved in the intervention arm 5 months after the intervention. The prevalence in the control arm was also reduced by 24% compared with baseline. The bed net use prevalence increased from 50% of the baseline up to approximately 90% in both arms, which should have contributed to the reduction even in the control arm. The result also suggests that standard-LLINs (OlysetNet in the present study) are still effective against a pyrethroid resistant vector population.^57^ At the least, standard-LLINs are able to reduce physical contact between humans and mosquitoes.

When considering the difference in rainfall before and after the intervention, the actual contribution of both LLIN products should be greater than these values. During the 3-month period before the first postintervention survey, the rainfall recorded was 289 mm, whereas the amount was 128 mm during the 3-month period before the baseline survey. Because a higher infection risk is usually associated with higher rainfall,^58^ the prevalence in the latter period would have been higher than the baseline prevalence of 60% if the intervention was not implemented. The rainfall was even higher (455 mm) during the 3-month period before the second postintervention survey, which may partially explain the slight increases of prevalence in both arms. Despite the increase of infection risk, the added effectiveness of PBO-LLIN was still clear in the second survey.

The added effectiveness of PBO-LLIN on Hb concentrations was also clearly recognized 12 months after the intervention, even though the difference was not apparent after 5 months. This result is comparable to that of the Tanzania study.^20^ In that study, the difference in anemia prevalence (Hb < 8 g/dL) between the PBO-LLIN and standard LLIN arms was not statistically significant 4 months after the net distribution, but it became significant after 9 and 16 months. On the other hand, the difference in anemia prevalence (Hb < 11 g/dL) was significant between the two groups 6 months after the net distribution in the Uganda study, but the effect was weaker later except in the areas where OlysetPlus was distributed.^21^ The inconsistency may be due to various factors, such as coinfection with schistosomiasis, which is particularly common in the present study area and the lake region of Uganda.^59,60^ Nevertheless, the results from the three studies confirmed that use of PBO-LLINs also improves anemia related to *Plasmodium* parasite infection.

Both entomological cross-sectional surveys clearly showed the impact of PBO-LLIN on the predominant vector species group, *An. gambiae* s.l., compared with standard LLIN. Despite the higher rainfall immediately before the second entomological survey, the density of *An. gambiae* s.l. was even lower in both arms compared with the first survey. The decrease of *An. gambiae* s.l. may be explained by a cumulative effect of both LLIN products despite increases of potential breeding habitats with the high rainfall. Before the present work, a study reported that nearly 90% of *An. gambiae* s.l. samples from the same sentinel survey were *An. arabiensis*.^27^ The proportion of *An. arabiensis* could be greater in the study area because PSC might underestimate the abundance of this exophilic species. The metabolic pyrethroid resistance is widespread in this species in the study area, but it does not have *kdr* resistance.^25^ An experiment with F1 progenies of field collected *An. arabiensis* from this study area showed that their susceptibility against permethrin increased 20-fold when PBO was topically added to their dorsal mesothorax.^26^ Although we did not confirm species of the anophelines collected during the study period, the previous studies suggest that a majority of *An. gambiae* s.l. samples were *An. arabiensis* with metabolic resistance.

In the case of *An. funestus* s.l., the difference in density between the two arms was not statistically significant in the first cross-sectional survey, but the density was significantly lower in the intervention arm in the second survey after 10 months of intervention. The density was apparently too small to produce a statistical significance for the difference between both arms in the first survey. An increased Lake Victoria water level may explain the greater numbers of *An. funestus* s.l. in the second survey. This species group exclusively inhabits swamps associated with the lakeshore of the present study area.^28,61^ The lake water level started rising from the middle of 2011, which could expand the potential habitat inland.^62^ Rainfall runoff from the surrounding mountains generally affects the lake water level, as well as activities of the Nalubaale hydropower dam. Consequently, the increase of *An. funestus* s.l. would be responsible for the slight increase of PCRpfPR in the second postintervention survey. The habitats of *An. funestus* s.l. mainly occur along the lakeshore in the northwestern part of the study area, and the abundance might vary among the clusters.^28^ In fact, the coastline of the control arm is approximately 4 km longer than that of the intervention arm (15 versus 11 km), and the baseline density of the control arm was higher. However, the two-stage analyses should have adjusted the imbalance.^49,50^ Nevertheless, the result from the second cross-sectional survey suggests that use of PBO-LLINs controlled the increase of this species group better than standard LLINs. The study in an adjacent area found that nearly all anophelines belonging to *An. funestus* s.l. were *An. funestus* s.s.^29^ PBO also enhances the susceptibility of this species to permethrin more than 20 times in the topical application assay.^26^ When considering both *An. arabiensis* and *An. funestus* s.s., these studies suggest that a metabolic resistant vector population likely predominates in the present study area.

On the other hand, the permutation tests produced a *P* value slightly > 0.05 when the adjusted differences in density between the two arms were tested for *An. gambiae* s.l. as well as anopheline in the postintervention sentinel survey. However, the CIs produced by bootstrapping were below 0 (negative) for the two taxonomic groups. The discrepancy is probably due to the difference in resampling (randomization) between the two methods. Although permutation produces a robust result for null hypothesis testing, the accuracy of bootstrapping becomes less for hypothesis testing with a small sample size.^56^ However, the latter is more suitable for estimating CIs. The discrepancy was little, and the results from both approaches still imply the added effectiveness of PBO-LLIN on *An. gambiae* s.l. and anopheline including *An. gambiae* s.l. and *An. funestus* s.l.

In the case of *An. funestus* s.l., the results from the postintervention sentinel survey were likely due to the low density. Although the number (*n* = 1,200) of PSCs was large, the number (*n* = 456) of *An. funestus* s.l. collected was apparently too small to produce a statistical significance. The low density was due to including dry periods in the longitudinal surveillance. Overall, the results of the statistical tests were consistent throughout the three entomological surveys when *An. gambiae* s.l. and *An. funestus* s.l. were combined. Along with the epidemiological results, this study confirms the effectiveness of PBO-LLINs in an area where a metabolic resistant vector population likely predominates.

### Limitation.

Compared with the previous cRCTs on PBO-LLIN, the present study area was smaller, which limits generalizing from the results. The small area size also limited the number of clusters to four for each arm, which is the minimum requirement for cRCT. The small number of clusters may increase the variability among them and increase a required cluster size. To overcome these issues, we applied the two-stage procedure.^50^ This method was able to balance the variability among the clusters adapting the baseline data and adjusted for fixed-covariates such as age and SES in the epidemiological analyses.^50^ Then we used permutation for null hypothesis testing which is fairly robust even if a sample size is small.^50^ When compared with unadjusted results, the adjusted results suggest the added statistical power.^49,50^ However, the small number of clusters might still produce the discrepancies between the *P* values with permutation and CIs with bootstrapping.^56^

On the other hand, the small area size allowed us to better control the study. For instance, we were able to fully control the net distribution by visiting door to door. With consent we removed nearly all nets already owned and replaced them with new nets to prevent a potential bias from mixing them, which apparently was not practiced in the previous cRCTs.^20,21^ Two PBO-LLIN products for testing were mixed within the same clusters in the Uganda study, and the distribution took almost 1 year before the first postintervention survey. Nevertheless, both studies were able to demonstrate the effectiveness of PBO-LLINs, likely because of the large numbers of clusters.

More than 25% of selected children were not found or did not show up at the testing centers. There might be several reasons for missing children. They might have migrated to other areas during the short period after the house survey. Parents and caretakers often change children’s names in this area, or children have a few names or nicknames, which makes it difficult for field assistants to identify them. Additionally, caretakers might have been too busy to take their children to the testing centers. The missing children would have reduced the statistical power to detect the effect, especially when the numbers of missing children significantly vary among the clusters. However, the effects due to missing children should have been minimized by randomizing the clusters. Because the analyses clearly revealed the effectiveness of PBO-LLINs, to a certain degree adjusting for the confounders such as SES should have resolved the potential bias related to missing children. However, if the missing children were different in a certain characteristic from the tested children, the epidemiological findings of the present study might not apply to them.

Despite the lower sensitivity of RDT, the overall RDTpfPR and PCRpfPR were almost the same (47% and 46%, respectively). False positives associated with RDT may explain this phenomenon. The RDT kit used in the present study targets *P. falciparum* specific histidine-rich protein 2 (HRP2), which may persist in the blood for a few weeks after treatments.^63,64^ In high malaria transmission areas, frequent reinfections and treatments increase RDT false positives. This phenomenon is particularly common among children who have not acquired immunity. The positive rates of eight RDT kits (ranged from 44% to 48%) were also similar to that of PCR (51%) in a comparative study in the lake region.^65^ In contrast, the RDTpfPR and PCRpfPR were 3% and 8%, respectively, in a comparative study in the highlands of western Kenya where transmission is usually low.^66^ The lower RDTpfPR in the highlands is likely due to a few false positives. Nevertheless, the present study had slightly higher RDTpfPRs than PCRpfPRs in some cases. This may be due to targeting only children in the present study. The comparative study in the lake region included adults who usually have a higher proportion of submicroscopic and asymptomatic infections than children.^65,67^ As submicroscopic infections increase, PCR detects more positive cases compared with RDT. Although the comparative study in the lake region did not use the same RDT kit as the present study, the spatial and temporal factors were less likely associated with the RDTpfPR, because all three study sites including the present study were conducted within a distance of 50 km and during a similar period. To minimize human errors in the field, two persons confirmed the RDT results in the present study. On the other hand, it is still possible that quality variations between different RDT products and lots produced the higher RDTpfPR.^68^ Despite the limitations of RDT, in the present study, the data of RDT still indicated the effectiveness of PBO-LLINs.

## CONCLUSION

Three cRCTs, including the present study, demonstrated the effectiveness of PBO-LLINs.^20,21^ The present study contributes to the growing evidence base, and provided two major outcomes: first, the superiority of PBO-LLIN over standard LLINs was confirmed in an area where the vector population with metabolic resistance likely predominates, and second, validation of a more accurate parasite detection method (PCR). The latter information is particularly important for planning control programs toward elimination in a lower transmission area where asymptomatic infections become main targets. Considering the comparable results from the three cRCTs, standard LLINs should be replaced with PBO-LLINs for strengthening the current malaria control programs. However, the cost-effectiveness of PBO-LLINs needs to be assessed.
